# Does Occupational Exposure of Shahid Dastghieb International Airport Workers to Radiofrequency Radiation Affect Their Short Term Memory and Reaction Time?

**Published:** 2015-09-01

**Authors:** S. Jarideh, S. Taeb, S. M. Pishva, M. Haghani, S. Sina, S. A. R. Mortazavi, M. A. Hosseini, S. Nematollahi, N. Shokrpour, M. Hassan Shahi, S. M. J. Mortazavi

**Affiliations:** 1 Ionizing and Non-ionizing Radiation Protection Research Center (INIRPRC), Shiraz University of Medical Sciences, Shiraz, Iran; 2 Shahid Dastghieb International Airport, Shiraz, Iran; 3 Radiation Research Center, Nuclear Engineering Department, School of Mechanics, Shiraz University, Shiraz, Iran; 4 Medical Student, School of Medicine, Shiraz University of Medical Sciences, Shiraz, Iran; 5 Department of Biostatistics, School of Medicine, Shiraz University of Medical Sciences, Shiraz, Iran; 6 School of Paramedical Sciences, Shiraz University of Medical Sciences, Shi¬raz, Iran; 7 Medical Physics and Medical Engineering Department, School of Medicine, Shiraz University of Medical Sciences, Shiraz, Iran

**Keywords:** Airport, Electromagnetic fields, Occupational Exposure, Cognitive Functions, Short Term Memory, Reaction Time

## Abstract

**Background:**

Airport workers are continuously exposed to different levels of radiofrequency microwave (RF/MW) radiation emitted by radar equipments. Radars are extensively used in military and aviation industries. Over the past several years, our lab has focused on the health effects of exposure to different sources of electromagnetic fields such as cellular phones, mobile base stations, mobile phone jammers, laptop computers, radars, dentistry cavitrons and MRI. The main goal of this study was to investigate if occupational exposure of Shahid Dastghieb international airport workers to radiofrequency radiation affects their short term memory and reaction time.

**Methods:**

Thirty two airport workers involved in duties at control and approach tower (21 males and 11 females), with the age range of 27-67 years old (mean age of 37.38), participated voluntary in this study. On the other hand, 29 workers (13 males, and 16 females) whose offices were in the city with no exposure history to radar systems were also participated in this study as the control group. The employees’ reaction time and short term memory were analyzed using a standard visual reaction time (VRT) test software and the modified Wechsler memory scale test, respectively.

**Results:**

The mean± SD values for the reaction times of the airport employees (N=32) and the control group (N=29) were 0.45±0.12 sec and 0.46±0.17 sec, respectively.  Moreover, in the four subset tests; i.e. paired words, forward digit span, backward digit span and word recognition, the following points were obtained for the airport employees and the control group, respectively: (i) pair words test: 28.00±13.13 and 32.07±11.65, (ii) forward digit span: 8.38±1.40 and 9.03±1.32, (iii) backward digit span: 5.54±1.87 and 6.31±1.46, and (iv) word recognition: 5.73±2.36 and 6.50±1.93. These differences were not statistically significant.

**Conclusion:**

The occupational exposure of the employees to the RF radiation in Shahid Dastghieb international airport does not have any significant detrimental effect on their reaction time as well as short term memory.

## Introduction

The health risks associated with the presence of the electromagnetic waves in daily life have attracted much attention [1]. In this respect, a major concern involves the microwave sources found everywhere from the kitchens to the communication control offices such as radar (Radio Detecting and Ranging) system control [2, 3].

Radar, as an object-detection system to reveal the position and speed of objects,  has many industrial and scientific applications [4]. Most radar systems emit short radio wave pulses in the range of microwaves, with a relatively long time interval. The re-radiated pulses from the targets are detected, and a measure of target distance is provided by the interval between the transmitted and received pulses. The power must be relatively high at the transmission site, therefore the employees working in the offices close to the radar control system are regularly exposed to the pulsed electromagnetic fields with high power and frequency [5].

Recently some studies have been conducted on the health effects of occupational exposure to military radar radiations. It has been shown that occupational exposure to radar radiation leads to changes in somatic symptoms, anxiety and insomnia, social dysfunction, and severe depression [6]. One also finds the reported risks such as increased incidence of hemolymphatic cancers [7], reduced fertility [8], increased level of DNA damage and chromatid breaks [9] that are caused in presence of such devices. On the other hand, there are some published papers reporting no evidence of increased risks reverent for the above-mentioned symptoms associated with the radar emission effects on the employees. For instance, in a large U.S. Navy cohort with radar exposure, testicular cancer mortality was found to be lower than expected in the group with the potential for high exposure [10]. In a 40-year controlled longitudinal Belgian study, no increase in the mortality in Belgians who were in close contact with RF energy from military radar was founded [11].

Over the past several years, our lab at the Ionizing and Non-ionizing Radiation Protection Research Center (INIRPRC) has performed extensive experiments on the health effects of exposure of animal models and humans to different sources of electromagnetic fields such as cellular phones [12-19], mobile base stations [20], mobile phone jammers [21], laptop computers [22], radars [13], dentistry cavitrons [23] and MRI [24, 25].

It was shown that occupational exposure to military radar radiations may decrease the reaction time in workers [5].

The aim of this study is to investigate and debate this scientific open question more profoundly, by providing more scientific evidences for the health hazards of exposure to radar microwaves. The effects of radar radiowaves on the short term memory and the reaction time of the employees working in control and approach tower of Shahid Dastghieb international airport of Shiraz, were investigated. 

## Methods

### Participants

The Shahid Dastghieb international airport, Fars, Iran, has two types of radar: a preliminary and a secondary radar with the frequency ranges of 2.7 -2.8 GHz and 1030-1090MHZ, respectively. The secondary radar is located at 500 meters away from the control and approach tower, the closest building to it compared to the other parts of the airport.

32 employees of control and approach tower including 21 males and 11 females, with the age range of 27-67 years old, and the mean age of 37.37±8.39 , participated voluntary in this study.

29 people, 13 male, and 16 female volunteers not working near the radar system were also participated in this study as the control group. 

Before testing, the demographic information of each control and approach tower employee, simply called employee in the whole paper, such as age, education and the working duration at that place, was recorded in a questionnaire.

By exploiting the VRT software and standard Wechsler questionnaire consisting of four subtests, i.e. paired words, forward digit span, backward digit span and word recognition, the reaction time as well as short term memory of the employees were recorded. Subsequently, the results were analyzed by T- test on a computer

### Memory Test

The employees were checked by the so-called modified Wechsler memory scale test. As mentioned before, this memory test has four subtests. After rehearsing the test with the participants, they were asked by an expert in our research team to repeat back a list of digits, which were spoken live-voice at a rate of about one digit per second. This study was approved by the Research Ethics Committee in the School of Medicine in Shiraz University of Medical Sciences and informed consent was obtained from all participants.

### Reaction time test

A modified version of the Bracy simple visual reaction time (VRT) test [26] developed in the center for research in radiation sciences (CRRS), Shiraz University of Medical Sciences, Shiraz, Iran was used for the reaction time test. First, the test tutorial and instructions were presented to the participants and they had some practice. At this point, they were asked to right click on a computer mouse as soon as a red square on the display was substituted by a green one. Each test was repeated 7 times in both real and sham exposure phases by each employee to have a better statics.

## Results

The demographic and occupational data of the participants are presented in Table 1. As indicated in the table, the mean age of the participants was 37.37 ± 8.39 years, with a range of 27-67 years old. In addition, 34.4% of employees worked less than 8 h per day (5 days/week) and 65.6% worked 9-12 hours per day (less than 5 days/week).

**Table 1:** The recorded demographic and occupational data of the employees participated in this study (n = 61).

	**case**		**control**	
	**Frequency (%)**	**Mean (SD)**	**Frequency (%)**	**Mean (SD)**
**Age**				
Male	N=21 (65.6)	37.81 (6.8)	N=13 (44.8)	40.30 (7.99)
Female	N=11 (34.4)	36.54 (0.111)	N=16 (55.2)	32.43 (7.26)
Total	N=32 (100)	37.37 (8.39)	N=29 (100)	35.96 (8.46)
**Work Hours/Day **				
≤ 8 hours	11 (34.4)			
9-12 hours	21 (65.6)			
**Work Days/Week **				
< 5 Days	21 (65.6)			
5 Days	11 (34.4)			
**Work Experience **				
≤ 24 months	0			
25-48 months	3 (9.37)			
49-120 months	11 (34.4)			
> 120 months	18 (56.25)			
Distance from Antenna ≤500	32			

Also, as to the working experiences in the airport, 9.37% (3 people) of the participants had worked for 2-4 years, 34.4% (11 people) of them for 5-10 years, and finally 56.25% (18 people) more than 10 years. It is worth mentioning again that the employees’ office was located at the distance of 500 meter from the secondary radar. The relationship between the working experience and the reaction time of the participants is shown in Table 2.

**Table 2:** The relationship between reaction time and the participants’ working duration in the airport

	**Frequency (# of samples)** **Reaction Time (sec)** **Airport employees**		**Total**
Work Experience	≤ 0.432 sec	> 0.432 sec	
< 14	24	17	41
≥14	7	11	18
Total	31	28	61

Chi-square P-value=0.134 > 0.01

According to the results shown in the table, the Chi-square test do not show any meaningful relationship between the duration of working in the tower and neither the reaction time (P-value> 0.01) nor the short- term memory of the employees. However, an inverse association between the short-term memory and the reaction time is observed.

The reaction time of the airport employees and the control group are compared in Table 3. Comparing the data obtained from the airport employees with those of the control group, one immediately observes that there is no substantial difference in the reaction times of both groups.

**Table 3:** Mean (± SD) value for reaction time of the airport employees and the control group.

**Age group (yr) **	**Airport employees** **Reaction time (sec)** ** (Mean± SD) ** **(# of participants)**	**Control Group ** **Reaction time (sec) ** **(Mean± SD) ** **(# of participants) **	**P-value**
20-30	0.40±0.39 (N= 6)	0.37±0.06 (N= 8)	0.201
31-40	0.42±0.08 (N= 19)	0.44±0.09 (N= 15)	0.398
41-50	0.55±0.19 (N= 5)	0.51±0.20 (N= 3)	0.831
>50	0.66±0.19 (N= 2)	0.76±0.34 (N= 3)	0.754
Total	0.45±0.12 (N= 32)	0.46±0.17 (N= 29)	0.791

Finally, as indicated in Table 4, the short-term memory of airport employees and the control group for all four subtests, paired words, forward digit span, backward digit span and word recognition, did not have any significant differences.

**Table 4:** Mean (± SD) values for the short term memory scores of the participants in each subtest, including forward and backward digit span, paired words and word recognition.

	**Airport employees ** **(Mean± SD)** **(n=32)**	**Control Group ** **(Mean± SD** **(n=29)**
Paired words	28.00±13.13	32.07±11.65
Forward digit span	8.38±1.40	9.03±1.32
Backward digit span	5.54±1.87	6.31±1.46
Word Recognition	5.73±2.36	6.50±1.93

## Discussion and Conclusion

The effects of exposure to radar microwaves, on the reaction time and the short term memory of the employees in the control and approach tower of the Shahid Dastghieb international airport are investigated in this study. 

The results of different tests indicated, there was no significant difference between the mean reaction time of the employees, and those of the control group of participants, 0.42±15 sec and 0.45±0.12 sec, respectively. Also, No significant differences are found in the score of the four subtests, including paired words, ordinal numbers, reverse numbers, and recognizing words for the two groups.

Since the employees’ office under the study was located only 500 m away from the secondary radar, emitting waves with frequency of 1030-1090 MHz; the electromagnetic, electric and magnetic field intensities at their office, 2.37 µw/m2, 12.7v/m and 0.2 mT respectively, were all lower than the standard thresholds. Consequently, it is concluded that occupational exposure has no effect on the short-term memory as well as the reaction time of the employees in international Shiraz airport. 

In our previous investigations the effects of the mobile phones’ radio waves were studied on the reaction time using VRT test [27, 28] together with the short-term memory examination by the Wechsler test.  According to the results of our investigation, the radio waves have significant impacts on the adults’ short-term memory [29]. The results showed a reduced reaction time for the students in Shiraz University of Medical Sciences with an age range of 18-30 years old [27]. However, we could not detect any reduced reaction times for 60 children with the age range of 8-10 years old [28]. Additionally, since the short term memory and the reaction time of the adult employees in the control and approach tower of Shahid Dastghieb international airport were not affected by radar radio waves, it may be concluded that the work enviroment is safe at Shahid Dastghieb international airport.

One must notice that the findings of this study are not consistent with the recent  published paper showing that the short-term memory and reaction time of the employees working close to the radar were obviously lower than those of the control group [5]. The discrepancy in the results returns back to both the frequency of the electromagnetic waves and the distance of the employees from the radar system.

In one of our previous studies [5], the employees were working at a distance of 4 meters away from a radar system with the frequency peak of 2 GHz. However, the Shadid Dastghieb airport employees work in an office located 500 meters away from the secondary radar emitting the waves with the frequency range of 1030-1090 MHZ.
